# DART: diagnostic-CT-enabled planning: a randomized trial in palliative radiation therapy (study protocol)

**DOI:** 10.1186/s12904-022-01115-y

**Published:** 2022-12-09

**Authors:** Melissa O’Neil, Timothy K. Nguyen, Joanna Laba, Robert Dinniwell, Andrew Warner, David A. Palma

**Affiliations:** Department of Radiation Oncology, London Health Sciences Centre, Western University, P.O. Box 5010, STN B, 800 Commissioners Rd. E, London, Ontario N6A 5W9 Canada

**Keywords:** Diagnostic CT planning, palliative radiotherapy, randomized controlled trial, Alternative radiation workflow

## Abstract

**Background:**

Palliative radiotherapy (PRT) is an effective treatment for managing symptoms of advanced cancer. At least half of all radiation treatments are delivered with palliative intent, aimed at relieving symptoms, such as pain or shortness of breath. Symptomatic patients must receive PRT quickly, therefore expeditious treatment planning is essential. Standard radiation planning requires a dedicated CT scan acquired at the cancer centre, called a ‘CT simulation’, which facilitates treatment planning (i.e. tumor delineation, placement of radiation beams and dose calculation). However, the CT simulation process creates a bottleneck and often leads to delays in starting treatment. Other researchers have indicated that CT simulation can be replaced by the use of standard diagnostic CT scans for target delineation and planning, which are normally acquired through the radiology department as part of standard patient workup.

The goals of this feasibility study are to assess the efficacy, acceptability and scalability of diagnostic-CT-enabled planning, compared to conventional CT simulation planning, for patients receiving PRT to bone, soft tissue and lung disease.

**Methods:**

This is a randomized, phase II study, with 33 PRT patients to be randomized in a 1:2 ratio between conventional CT simulation (Arm 1), and the diagnostic CT enabled planning workflow (Arm 2). Patients will be stratified by treatment target volume (bone and soft tissue metastasis vs. primary or metastatic intrathoracic disease targets).

The primary endpoint is the amount of time the patient spends at the cancer centre. Secondary endpoints include efficacy (rate of plan deliverability and rate of plan acceptability on blinded dose distribution review), stakeholder acceptability (based on patient and clinician perception of acceptability questionnaires) and scalability.

**Discussion:**

This study will investigate the efficacy, acceptability and scalability of a “sim-free” PRT pathway compared to conventional CT simulation. The workflow may provide opportunity for resource optimization by using pre-existing diagnostic imaging and requires minimal investment due to its similarity to current PRT models. It also offers potential benefit to patients by eliminating an imaging procedure, reducing the amount of time spent at the cancer centre, and expediting time to treatment.

**Trial registration:**

Clinicaltrials.gov identifier: NCT05233904. Date of registration: February 10, 2022; current version: 1.4 on April 29, 2022.

**Supplementary Information:**

The online version contains supplementary material available at 10.1186/s12904-022-01115-y.

## Background

Palliative radiotherapy (PRT) is an effective and efficient intervention in managing the symptoms of advanced cancer [1]. At least half of all radiation treatments are delivered with palliative intent [2], and symptom relief is reportedly experienced by 60-80% of recipients [3]. Treatment indications are broad, but localized pain from bone (and soft tissue) metastases and thoracic PRT for dyspnea and airway preservation are the most frequent indications and are estimated to account for 35-40 and 25% of all courses respectively [4, 5].

The PRT workflow typically involves a radiation oncology (RO) consultation, CT simulation appointment, treatment planning, quality assurance (QA), and treatment delivery. 3D CT-simulation for target delineation and dosimetry is considered the gold standard [6] in most radiotherapy departments and represents a major checkpoint in the patient care pathway.

Expediency is also a key component of PRT [7]. The implementation of rapid access PRT programs has streamlined the referral-to-treatment pathway, reduced wait times, decreased the number of required hospital visits, and resulted in improved patient satisfaction [8–11]. However, these newer models of care still depend upon a conventional workflow, a process that usually takes at least 4 hours and translates into significant wait times, in some cases even dissuading patients from receiving same-day service. This model also requires reserved or readily available CT-simulation appointments, which can be a scheduling challenge. In a local context, strategic resource planning for decommissioning and replacement of two CT-simulators has underscored the need to explore resource optimization.

Such efforts are not only relevant to short-term pressures resulting from machine replacement, but also because demands on the cancer care system and radiation programs are only anticipated to increase. Cancer is the leading cause of death in the province, nearly one out of every two Ontarians will develop cancer in their lifetime, and incidence is only expected to rise as the population ages [12]. Additionally, as treatments improve, many patients live longer and require more PRT courses than patients in the past (the number of people treated with radiation has grown by about 2% per year since 2012). Sustainability will not only depend on managing existing resources, but on optimizing them through innovative models of care.

Several authors [10, 13–19] have explored alternatives to the conventional PRT pathway, and specifically opportunities to obviate the need for CT-simulation. Wong R and colleagues [13] demonstrated the dosimetric validity and clinical feasibility of using cone beam CT (CBCT) acquired on the treatment unit to plan PRT treatments. This workflow successfully eliminates CT-simulation but still entails significant “on-bed” times for patients (ranging from 30 to 50 minutes), requires seamless and synchronous multidisciplinary team (MDT) collaboration, and “pressurizes” the QA process. Patient satisfaction in the CBCT pathway was reported as “equivalent,” but there was no control group and no citation of conventional PRT satisfaction. The most common patient complaint was having to remain motionless on the treatment bed for the duration of the appointment (mean ± SD time of 32.7 ± 4 minutes). The workflow has since been discontinued due to challenges with scheduling and RO availability.

Wong S and colleagues [10] propose an alternative workflow, which utilizes recent diagnostic CT (dCT) scans. In this sequential 2-phase investigation, the authors confirmed dosimetric validity and demonstrated efficacy in a prospective cohort of 30 PRT patients. Participants still underwent CT-simulation/planning to ensure a “back-up” plan was available, but the authors reported 100% deliverability with dCT plans and dosimetric variation consistent with similar investigations [13, 14].

Using the workflow described above, Schuler and colleagues [20] subsequently investigated pain response for 160 PRT patients using electronic patient-reported outcomes. Given that only the planning image dataset had changed (from CT simulation to diagnostic-CT), they hypothesized and confirmed that overall and complete pain response rates were equivalent to published evidence [21].

This proposed feasibility study will evaluate a diagnostic-CT-enabled planning workflow and assess efficacy, acceptability and scalability. The workflow will employ CBCT-planning as a back-up pathway and incorporate optical surface guidance for ease of set-up reproducibility. The study will also expand eligibility criteria from previous work to include thoracic disease targets in addition to bone and soft tissue disease. Pain response or treatment response were not chosen as endpoints given previous evidence of equivalency and/or the reasonable assumption of equivalency as both arms of the investigation ultimately receive the same treatment. The only difference between arms is the planning image set. Thus, the evaluation of the efficacy of the diagnostic-CT-enabled planning workflow, in this context, is dependent upon the rate of plan deliverability (i.e. the experimental planning arm produced a deliverable plan) and acceptability (i.e. the experimental planning arm produced a plan determined to be acceptable as per institutional guidelines).

## Objectives

To assess the efficacy, acceptability and scalability of diagnostic-CT-enabled planning, compared to conventional CT simulation planning, for patients receiving PRT to bone, soft tissue and lung disease.

*Research Hypothesis: Diagnostic-CT-enabled planning for PRT patients is an effective, acceptable and scalable alternative to CT-simulation.*

### Endpoints

#### Primary endpoints

The primary endpoint is time in centre (TIC) on the treatment day. This is defined as the total time in hours spent at the cancer centre from the scheduled CT simulation (Arm 1) or treatment delivery (Arm 2) appointment until beam delivery completion.

#### Secondary endpoints

##### Efficacy

- Rate of plan deliverability
- Rate of plan acceptability on blinded review of dose distributions

##### Acceptability

- Stakeholder evaluation (patients, ROs, medical radiation therapists (MRTs), physicists) using a Likert scale modeled on the Theoretical Framework of Acceptability (version 2) [22]

##### Scalability

- Completion of the Intervention Scalability Assessment Tool (ISAT) [23] by stakeholders for submission to program leads

## Study design

This will be a pragmatic, randomized, controlled, open-label, feasibility trial.

All study activities will take place at the London Regional Cancer Program in London, Ontario, Canada.

Patients will be randomized in a 1:2 ratio between the current standard treatment workflow (Arm 1) and the experimental treatment workflow (Arm 2).

Patients will be stratified by treatment target volume. Group 1 will include bone and soft tissue metastasis targets, and Group 2 will include primary or metastatic intrathoracic disease targets.

## Patient selection

Potential study participants may be identified/screened by a member of the study team or any staff RO, and patients who meet inclusion/exclusion criteria will be eligible for recruitment. Potential participants will receive a verbal explanation of the study by a study team member/RO, receive a paper Letter of Information/Consent, and be given the opportunity to discuss the trial/ask questions with the Collaborating Investigator.

Potential participants who feel comfortable signing consent at this initial encounter will be allowed to do so, but reminded of their right to withdraw their consent at any time and for any reason.

Potential participants who would like additional time to review the LOI and/or discuss things with their care partners will be encouraged to do so. This additional consideration time is clinically reasonable given that these patients would generally receive treatment within 7-14 days of consult, and because any oncologic emergency requiring immediate intervention would exclude the patient from participation. Potential participants will be informed that the Collaborating Investigator will follow-up with them via telephone within 2-3 business days, but that they are free to contact the Collaborating Investigator sooner if they make a decision. Those who wish to participate will be given the option to complete an electronic consent form prior to their first radiation appointment, or to complete the form with the Collaborating Investigator at a separate appointment (scheduled in advance of the treatment day).

### Inclusion criteria

#### Patient inclusion criteria

- Age 18 years or older
- Able to provide informed consent
- Patient has consented to PRT to bone/soft tissue metastases or primary targets in the thorax, abdomen or pelvis and RO will use simple planning techniques (i.e. parallel-opposed pair or direct field beam arrangement)
- Patient will be scheduled for same day simulation and treatment (if randomized to Arm 1)
- Patient has a pre-existing and recent (i.e. within 4 weeks of time of enrollment) diagnostic CT or CT-fused scan with full visualization of the region-of-interest which has been acquired from an approved diagnostic scanner (CT scans must have been performed within 4 weeks [28 days] of the date of trial enrollment to minimize the risk of significant interval radiographic change [s]).
- Patient positioning for diagnostic scan is deemed acceptable and reproducible (e.g., patient is lying supine and relatively flat, there is no/minimal motion blur, etc.)
- Intravenous (IV)/oral contrast in the region-of-interest is permitted as long as it does not create artifact which obscures the target volume (density override calculations may be required)

#### Clinician inclusion criteria

- Age 18 years or older
- Able to provide informed consent
- Participation in the workflow of an arm 2 patient in the capacity of Radiation Oncologist, Medical Physicist, or Medical Radiation Technologist (Therapy)

### Exclusion criteria

#### Patient exclusion criteria

- Any contraindication to receiving radiation
- Oncologic emergencies and/or on-call cases
- Pregnant or lactating women
- Cases requiring composite dosimetric planning to account for previous radiotherapy or extended distance set-up
- Any contraindication to receiving radiation

#### Clinician exclusion criteria

- None

## Pre-treatment evaluation

- Eligibility according to inclusion and exclusion criteria
- History and physical examination

## Treatment schema

**Fig. 1:**
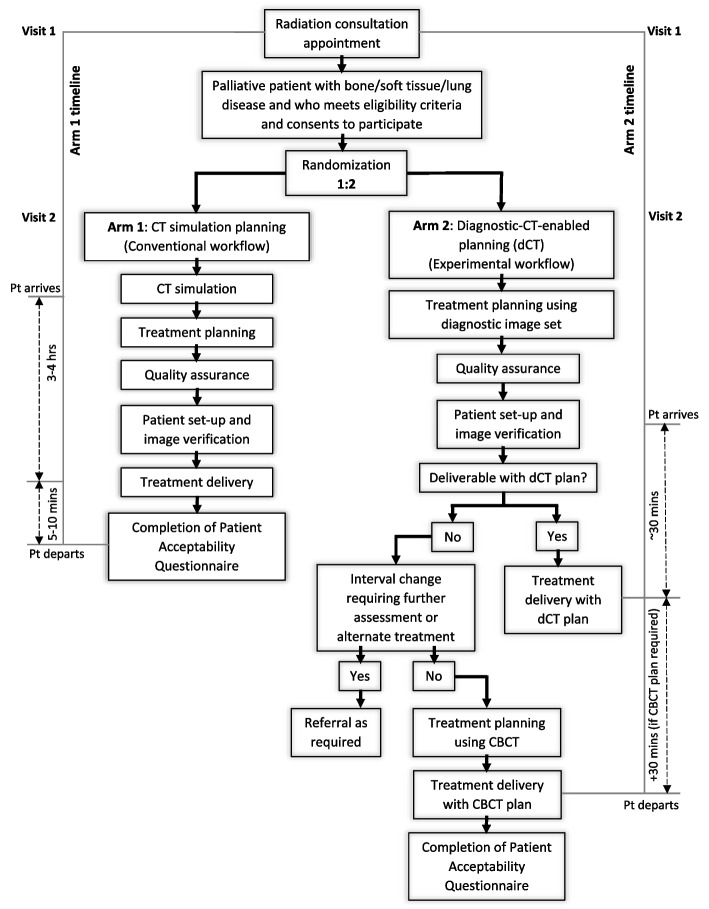
DART trial schema

Please see Fig. 1. For detailed treatment planning and delivery information, please see Section 8.

## Registration and randomization

All study data will be entered into REDCap; an electronic case report form database. De-identified supporting source documents will be uploaded directly into REDCap.

### Registration and randomization procedure

- Login to REDCap, add a new record and a study number will be automatically generated.
- Complete the Enrollment Form in REDCap. Upload the de-identified consent form and signed Statement of Eligibility.
- If the participant meets all the eligibility criteria, complete the Randomization form.

## Treatment plan

The electronic booking action form (EBAF) will be completed *after* randomization. ROs must specify “DART” and indicate the trial arm to facilitate bookings. For Arm 2 patients, the EBAF must specify the diagnostic image set to be imported for planning.

### Arm 1: conventional workflow

#### Scheduling

Patients will be booked for CT simulation and treatment as per the local institution’s standard practice.

#### CT simulation

As per local standard practice.

#### Target/organ-at-risk contouring

ROs must generate, at minimum, a planning target volume (PTV) contour. Organs-at-risk (OARs) may be contoured at the RO’s discretion to guide multileaf collimators (MLC) shielding, but optimization objectives are not allowed.

#### Dose and fractionation

All palliative dose/fractionation schemes are eligible to a maximum total dose of 30 Gy and a maximum of 10 fractions.

#### Planning and QA

Patients in Arm 1 will undergo simple palliative planning and QA as per the local institution’s standard practice. Plans will include no more than two fields and will not employ beam modification devices aside from MLCs.

#### Treatment delivery

##### Immobilization and set-up

As per local standard practice.

##### Image guided radiotherapy

*A CBCT must be performed for image-guidance at fraction 1*. Subsequent image-guided radiation therapy (IGRT) is at the discretion of the treating RO/radiation therapists in the context of local imaging protocols.

### Arm 2: experimental workflow

#### Scheduling

Patients randomized to Arm 2 *do not* require a CT simulation appointment. A radiation treatment appointment will be scheduled on an optical surface guidance-equipped treatment unit as soon as available, but a minimum of 24 hours is required between EBAF processing and fraction 1. Arm 2 patients must be scheduled between 10:00 am and 4:00 pm. The treating RO must be on-site and available by pager during the treatment appointment.

#### CT simulation

CT simulation is not required, but the remainder of the patient care pathway, including CT-related activities, will still apply. Instead of a simulation, CT staff will import the specified diagnostic image set and then complete tasks as per standard practice.

#### Target/organ-at-risk contouring

ROs must generate, at minimum, a PTV contour. OARs may be contoured at the RO’s discretion to guide MLC shielding, but optimization objectives are not allowed.

#### Dose and fractionation

All palliative dose/fractionation schemes are eligible to a maximum total dose of 30 Gy and a maximum of 10 fractions.

#### Planning and QA

Patients in Arm 2 will also undergo simple palliative planning and QA as per the local institution’s standard practice. Plans will include no more than two fields and will not employ beam modification devices aside from MLCs.

Treatment unit QA will include importing an external contour DICOM structure into VisionRT.

#### Treatment delivery

##### Immobilization and set-up

A full-body Vaclok bag, which has been shaped to replicate the curve of diagnostic machine couch tops, is required. Using optical surface guidance, unit therapists may use available pillows, cushions, headrests and other passive immobilization devices as required to closely (within a predefined tolerance) reproduce patient set-up in the region-of-interest. Once an acceptable set-up has been achieved, unit therapists will document the set-up instructions in the electronic chart. Optical surface guidance is to be used for subsequent set-ups, though non-permanent skin markings are also allowed.

##### Image guided radiotherapy

*A CBCT must be performed for image-guidance at fraction 1*. A surface reference must be captured after shifts have been applied. Subsequent IGRT is at the discretion of the treating RO/radiation therapists in the context of local imaging protocols.

The treating RO, clinical specialist radiation therapist, and/or medical physicist should be paged to review the image match if there are any concerns.

##### Undeliverable plan

Plans may be deemed undeliverable in the following scenarios:

- The treatment position could not be reproduced within tolerance *and* there is significant anatomical or external contour variation that cannot be corrected with IGRT
- Progression has occurred such that the target volume requires expansion for adequate coverage
- Changes are observed on CBCT that contraindicate PRT
- CBCT reveals new findings such as interval development of pathological fracture, atelectasis, etc. which warrant PRT deferral for further assessment or involvement of other services

At the discretion of the RO, CBCT-enabled planning may be enacted if a diagnostic-CT-enabled plan is considered undeliverable. This process is described in the next section.

##### Treatment delivery with CBCT plan

If the treating RO decides to engage this process, a DART-team medical physicist will be paged to the unit immediately to import/register/prepare the CBCT acquisition for planning. While the plan is generated, the patient can stay on the treatment bed or wait in the unit waiting area. The RO will re-contour the target on the CBCT and delineate field borders/shielding. The medical physicist will generate a simple plan for immediate RO approval and perform the required physics QA. A unit therapist will then perform new case QA. The patient can then be treated using the CBCT plan after repeat CBCT for verification.

## Subject discontinuation/withdrawal

Subjects may voluntarily discontinue participation in the study at any time. If a subject is removed from the study, the clinical evaluations that would have been performed at the end of the study should be obtained.

## Data collection and evaluation of efficacy, acceptability, and scalability

### Efficacy

#### Primary endpoint: time in centre (TIC)

TIC is defined as the total time in hours spent at the cancer centre and will be measured as follows:

- *Arm 1*: Scheduled CT simulation appointment time to time of beam delivery completion.
- *Arm 2*: Scheduled radiation treatment appointment time of beam delivery completion.

If machine/technical/patient-related delays or other unforeseen circumstances cause a delay to appointment time of more than 15 minutes, start time will be recorded and an adjusted TIC will be calculated based on the actual appointment start time. The intention of the adjusted TIC is accurate capture of TIC under normal operating conditions.

#### Plan deliverability

Plan deliverability will be reported as either yes or no. *Primary Plan Deliverability* will refer to the deliverability of the diagnostic-CT-enabled treatment plan. *Secondary Plan Deliverability* will refer to the deliverability of the CBCT-based plan in cases where a CBCT-based plan is generated.

For any undeliverable plan, the RO must document the reasons why the plan was undeliverable and the PI must be alerted within 24 hours.

#### Plan acceptability

Plan acceptability will be determined by blinded RO review, performed by an expert panel comprised of RO members from the local Palliative Disease Site Team. Because a review of the *planned* dose distribution would not preserve blinding due to visibly apparent characteristics of CT/CT simulation imaging, plan acceptability will be assessed by review of dose distribution of the *delivered* plan shown on the fraction 1 image matching CBCT.

Plan acceptability is defined according to the following local criteria for simple palliative planning:

- *For parallel-opposed pair plans:*
  - Adequate (as per RO discretion) coverage of the target volume (PTV) by the 90% isodose line
  - Hot spot (maximum point dose) is ≤115% of prescribed dose OR if hot spot is > 115% then the total volume of the hot spot should be <2cm^3^
- *For single direct field plans:*
  - Adequate (as per RO discretion) coverage of the target volume (PTV) by the 80% isodose line
  - Hot spot (maximum point dose) is ≤125% of prescribed dose OR if hot spot is > 125% then the total volume of the hot spot should be <2cm^3^

Blinded-reviewers will ascribe a score of 1, 2 or 3 to each plan based on the following criteria:

1. Acceptable
2. Minor deviation but still acceptable
3. Unacceptable – plan deficiency may impact clinical outcome

Plan acceptability *does not* refer to clinical judgements such as target delineation, beam arrangement, energy, field borders, shielding, dose/fractionation, etc.

### Stakeholder acceptability

Acceptability of the intervention will be evaluated by patients and clinicians using a Likert scale modeled on the Theoretical Framework of Acceptability (version 2) [22]. These questionnaires have been reviewed for face validity by an expert panel including ROs, medical physicists, and radiation therapists. See Additional file 1: Appendix A for Questionnaire Items based on the Theoretical Framework of Acceptability.

#### Patient acceptability

Patient acceptability will be assessed immediately following treatment delivery.

#### Clinician acceptability

Clinical acceptability will include all those involved in the treatment pathway and will be assessed within 5 working days of treatment delivery *for Arm 2 patients only*. Clinicians may be asked to complete the questionnaire up to 3 times to inform analysis as to whether perceptions change over time, for example, with increasing familiarity/exposure to the novel workflow.

Involved clinicians will be identified by the Collaborating Investigator or a member of the Lawson Health Research Institute Clinical Research Unit based on the electronic care path/treatment record.

On a clinician’s first encounter, an electronic version of the Clinician LOI/Consent will be emailed to their institutional email address. All staff will be aware of the possibility of receiving the LOI/Consent as they will be informed prior to opening the study at a staff meeting/inservice and by an email from the Principal and Collaborating Investigator. If/when the e-consent is completed and received, an e-version of the questionnaire will then be sent to the clinician.

Clinicians will be required to indicate how many times they have previously participated in the dCT-enabled treatment planning workflow on the questionnaire, but will only be required to complete one e-consent form. They will be advised of their right to withdraw participation at any time and for any reason, and that completing one questionnaire does not obligate them to complete further questionnaires.

### Scalability

Scalability will be determined by the outcome of program leadership’s evaluation of the completed ISAT [23]. The ISAT will be completed by stakeholders involved in the provision of the Arm 2 workflow and will include representation from RO, Medical Physics, Radiation Therapy (CT and Treatment Delivery) and Trial Investigators. The assessment will be submitted to program leadership and rated for scalability. Possible recommendations include: (i) Merits scale up, (ii) Promising, but further information/planning is warranted, and (iii) Does not merit scale up.

For the purpose of this trial, “scaling up” would include dosimetric calibrations of regional affiliate’s diagnostic imaging machines to enable expanded patient eligibility.

## Statistical considerations

### Randomization

Patients will be randomized in a 1:2 ratio between the current standard treatment workflow (Arm 1) and the experimental treatment workflow (Arm 2). This will be performed using permuted block design with block size known only to statistician until analysis is completed. The randomization sequence is known only to the statistician and uploaded into a restricted-access database (REDCap) housed on secure hospital servers at LHSC. Upon enrollment of a patient, the database will be accessed by the trial co-ordinator or collaborating investigator to obtain the next intervention in the random sequence, which will then be assigned to the patient.

### Sample size calculation

Based on a review of recent same-day treatments at the London Regional Cancer Program (LRCP), the estimated TIC in the standard arm of this trial will be 4.8 hours, with a standard deviation of 2. We hypothesize that in the experimental arm, the estimated TIC will be 2.5 hours, with the same standard deviation. Using a two-sided, two-sample t-test, with a 1:2 randomization, alpha of 0.05 and power of 80%, adjusting for 10% dropout, 33 patients are required, 11 in the standard arm and 22 in the experimental arm.

### Statistical analysis plan

Patients will be analyzed in the groups to which they are assigned (intention-to-treat). Comparisons between treatment arms for TIC (primary endpoint) will be performed using the two-sample t-test. TIC data is anticipated to be normally distributed. In the event such data is not normally distributed, non-parametric testing using the Wilcoxon rank sum test will be substituted as appropriate. For the secondary endpoints for efficacy (rate of plan deliverability and rate of plan acceptability on blinded review of dose distributions) and acceptability (stakeholder evaluations based on Likert scale), differences between treatment arms will be compared using the Chi-square test or Fisher’s exact test as appropriate. For the secondary endpoint for scalability based on the ISAT, differences between treatment arms will be compared using the two-sample t-test or substituted with Wilcoxon rank sum test if such data is not normally distributed, similar to primary endpoint.

## Adverse events

Arm 2 is not trialling a new treatment technique or dose/fractionation schedule and it is not anticipated to be associated with a higher risk of an adverse event or serious adverse event. As such, adverse event data will not be collected.

## Risks of participation

### Patient participant risks

Participation in this study does not increase the risk of experiencing the side effects of palliative radiation. Subjects in both study groups will receive the *same* type of radiation treatment. The *only difference* between groups is the type of CT imaging used to plan the treatment (CT simulation or diagnostic CT).

### Clinician participant risks/disadvantages

There are no anticipated risks related to participation as a clinician. Disadvantages for clinicians may include the time it takes or the inconvenience experienced in completing the questionnaire. Clinicians will not be compensated for their time, and completion of the survey will have no effect on employment, promotions, etc.

## Benefits

### Patient benefits

There is no guarantee of benefit in participating in this study. However, participants in Arm 2 may spend less time at the cancer centre than those in Arm 1.

### Clinician benefits

There is no guarantee of benefit in participating in this study, and completion of the survey will have no effect on employment, promotions, etc.

## Limitations

The investigation is non-blinded and participants will learn of both arms of the experiment during the consent process. This could introduce bias, specifically in relation to the perceived amount of time/effort required to participate (i.e. burden). A similar bias could manifest in the provider group as clinician’s who accrue may be inherently more enthusiastic about the novel workflow.

A qualitative approach using semi-structured interviews or focus groups would likely be a more comprehensive approach to assessing stakeholder acceptability. However, given the nature and timescale of the work, a quantitative, close-questioned survey, designed by the author and based on the TFA, was felt to be sufficient. Though the questionnaires will be reviewed by an expert panel of clinician-scientists for face-validity and by the appropriate Research Ethics Boards, the instrument will not undergo external validation.

While the phase II RCT design proposed herein will be limited in its degree of realizable internal validity compared to a highly powered phase III trial, it reasonably scaled to evaluate efficacy within a dissertation timeframe. Methods such as a structured protocol and carefully selected endpoints will allow for reliable and impactful statistical analysis, and randomization will provide rigor beyond a confirmatory cohort study and enable assessment of other facets of feasibility.

Convenience sampling will also limit the generalizability of results to larger/more heterogeneous populations. However, as eligibility is based on the type of radiation treatment participants will receive versus personal or disease characteristics, and as eligibility criteria are inclusive of many PRT patients, convenience sampling remains appropriate in this design.

## Ethical considerations

### Institutional review board (IRB) / research ethics board (REB)

The protocol (and any amendments), the informed consent form, and any other written information to be given to subjects will be reviewed and approved by a properly constituted Institutional Review Board (IRB)/Research Ethics Board (REB), operating in accordance with the current federal regulations (e.g., Canadian Food and Drug Regulations (C.05.001); US Code of Federal Regulations (21CFR part 56)), ICH GCP and local regulatory requirements. A letter to the investigator documenting the date of the approval of the protocol and informed consent form will be obtained from the IRB/REB prior to initiating the study.

### Informed consent

The written informed consent form to be provided to potential study subjects should be approved by the IRB/REB and adhere to ICH GCP and the ethical principles that have their origin in the Declaration of Helsinki. The investigator is responsible for obtaining written informed consent from each subject prior to beginning any study procedures and treatment(s). The investigator should inform the subject of all aspects of the study, including the potential risks and benefits involved. The subject should be given ample time and opportunity to ask questions prior to deciding about participating in the study and be informed that participation in the study is voluntary and that they are completely free to refuse to enter the study or to withdraw from it at any time, for any reason.

The informed consent must be signed and dated by the subject and by the person who conducted the informed consent discussion. A copy of the signed and dated written informed consent form should be given to the subject. The process of obtaining informed consent should be documented in the patient source documents.

### Confidentiality of subject records

The names and personal information of study participants will be held in strict confidence. All study records (case report forms, safety reports, correspondence, etc.) will only identify the subject by initials and the assigned study identification number. The investigator will maintain a confidential subject identification list (Master List) during the course of the study. Access to confidential information (i.e., source documents and patient records) is only permitted for direct subject management and for those involved in monitoring the conduct of the study (i.e., Sponsors, CRO’s, representatives of the IRB/REB, and regulatory agencies). The subject’s name will not be used in any public report of the study.

### Participant discontinuation/withdrawal

Subjects may voluntarily discontinue participation in the study at any time and for any reason. If a subject wishes to withdraw their information, they may contact any member of the study team to do so up until data analysis is undertaken.

### Interim analysis/early trial discontinuation

The trial team will conduct an interim analysis once the 10th patient is accrued. If the rate of plan deliverability in arm 2 is less than 60%, the team can, at its discretion, recommend cessation of the trial or exclusion of certain treatment sites that are deemed to be high-risk for plan undeliverability. The team may also recommend increasing or decreasing the target accrual in order to maintain statistical power.

## Authorship and dissemination

Upon completion of this project, the results will be published in a peer-reviewed journal and presented at one or more conferences.

Authorship will be decided by the principal investigators, and will be commensurate with the relative accrual and individual contribution of each investigator.

The results of this study will also comprise a master’s dissertation affiliated with and submitted to Sheffield Hallam University in the UK.

### Data sharing agreement

De-identified participant data from this trial will not be shared publicly. However, the full protocol will be published, along with the primary analysis of the outcomes.

## Financial support

This trial is funded by a Clinician-Scientist grant from the Ontario Institute for Cancer Research. The funding agency is not directly involved in data collection or analysis.

## Discussion

Palliative radiotherapy (PRT) is an effective means of managing advanced cancer symptoms and accounts for at least half of all radiation treatments. Expediency is a key component in this patient population, and prior research suggests that diagnostic-CT-enabled radiation treatment planning may be a viable and outcome-equivalent alternative to CT-simulation based workflows.

This is a randomized, phase II study, with 33 PRT patients randomized in a 1:2 (Arm 1: Arm 2) ratio will assess the efficacy, acceptability and scalability of diagnostic-CT-enabled planning, compared to conventional CT simulation planning, for patients receiving PRT to bone, soft tissue and lung disease.

The primary endpoint is the amount of time the patient spends at the cancer centre, and secondary endpoints include rate of plan deliverability, rate of plan acceptability on blinded dose distribution review, stakeholder acceptability and scalability. The workflow provides opportunity for resource optimization by using pre-existing diagnostic imaging, and requires minimal additional investment. It also offers potential benefit to patients by eliminating an imaging procedure and by reducing time spent at the cancer centre while awaiting treatment.

From a local perspective, Ontario cancer centres should give operational consideration to ensuring compensation for the Arm 2 workflow from Cancer Care Ontario’s (CCO) Quality-Based Procedure (QBP) funding. In the current funding model, centres are reimbursed for palliative treatment courses only when CT simulation is performed. Because Arm 2 uses a diagnostic image set instead of a CT simulation, a temporary trial QBP code was required to ensure reimbursement from CCO to the individual cancer centre, and this will be re-addressed upon completion of this study.

Another practical consideration in implementing the Arm 2 workflow is reserving the required “downstream” resources when CT simulation does not occur. At many centres, ours included, the number of patients requiring CT simulation is used to calculate the number of multidisciplinary hours required to prepare, plan, perform QA and deliver the radiation plan. Similarly, many centres use CT simulation throughput metrics to determine and calculate treatment unit availability for new patients. As a result, new methods of monitoring workflow in the absence of a CT simulation are being developed as part of this trial.

## Supplementary Information

**Additional file 1: Appendix A.** Questionnaire Items Based on the Theoretical Framework of Acceptability.

## Data Availability

Individual patient data will not be shared; ethics approval has not been sought or granted for raw data sharing.

## Abbreviations

CBCT: Cone-beam computed tomography
CCO: Cancer Care Ontario
CT: Computed tomography
DICOM: Digital Imaging and Communication in Medicine
EBAF: Electronic booking action form
ICH GCP: International Conference on Harmonisation-Good Clinical Practice
IGRT: Image guided radiotherapy
IRB: Institutional Review Board
ISAT: Intervention Scalability Assessment Tool
IV: Intravenous
LHSC: London Health Sciences CentreIS
LOI: Letter of Information
LRCP: London Regional Cancer Program
MDT: Multidisciplinary team
MLC: Multi-leaf collimator
MRT: Medical Radiation Therapist
PI: Principal Investigator
PRT: Palliative radiotherapy
PTV: Planning target volume
QA: Quality assurance
QBP: Quality-based procedures
RCT: Randomized controlled trial
REB: Research Ethics Board
REDCap: Research Electronic Data Capture
RO: Radiation Oncologist
SD: Standard deviation
TIC: Time in centre

## References

[1] Lutz S, Jones J, Chow E. Role of radiation therapy in palliative care of the patient with cancer. J Clin Oncol. 2014. 10.1200/JCO.2014.55.1143. 10.1200/JCO.2014.55.1143 PMC4152720 25113773

[2] Wu S, Singer L, Boreta L, et al. Palliative radiotherapy near the end of life. BMC Palliat Care. 2019. 10.1186/s12904-019-0415-8. 10.1186/s12904-019-0415-8 PMC6431041 30904024

[3] Samant R, Gooi A (2005). Radiotherapy basics for family physicians: a potent tool for symptom relief. Can Fam Physician.

[4] Spencer K, Morris E, Dugdale E, et al. 30 day mortality in adult palliative radiotherapy – A retrospective population based study of 14,972 treatment episodes. Radiother Oncol. 2015. 10.1016/j.radonc.2015.03.023. 10.1016/j.radonc.2015.03.023 PMC4504022 25861831

[5] Spencer K, Parrish R, Barton R, et al. Palliative radiotherapy. BMJ. 2018. 10.1136/bmj.k821. 10.1136/bmj.k821 PMC5865075 29572337

[6] Lachance C, McCormack S (2019). Magnetic resonance imaging simulators for simulation and treatment for patients requiring radiation therapy: A Review of the Clinical Effectiveness, Cost-Effectiveness, and Guidelines.

[7] Thavarajah N, Wong K, Zhang L, et al. Continued success in providing timely palliative radiation therapy at the Rapid Response Radiotherapy Program: a review of 2008-2012. Curr Oncol. 2013. 10.3747/co.20.1342. 10.3747/co.20.1342 PMC3671027 23737690

[8] Job M, Holt T, Bernard A. An evaluation of an advanced practice role in palliative radiation therapy. J Med Radiat Sci. 2019. 10.1002/jmrs.318. 10.1002/jmrs.318 PMC6545471 30809974

[9] Dennis K, Harris G, Kamel R, et al. Rapid Access Palliative Radiotherapy Programmes. J Clin Oncol. 2020. 10.1016/j.clon.2020.08.002. 10.1016/j.clon.2020.08.002 32826132

[10] Wong S, Roderick S, Kejda A, et al. Diagnostic Computed Tomography Enabled Planning for Palliative Radiation Therapy: Removing the Need for a Planning Computed Tomography Scan. Pract Radiat Oncol. 2021. 10.1016/j.prro.2020.10.010. 10.1016/j.prro.2020.10.010 33186781

[11] Rozanec N, Lavergne C, Harnett N. A Canadian experience of palliative advanced practice radiation therapy TIPS: Training, implementation, practice and sustainability. J tipsRO. 2021. 10.1016/j.tipsro.2021.01.003. 10.1016/j.tipsro.2021.01.003 PMC8110943 34007913

[12] Ontario Cancer Plan 5: 2019-2023. Cancer Care Ontario website. Accessed 14 July 2021. https://www.cancercareontario.ca/en/cancerplan.

[13] Wong R, Letourneau D, Varma A, et al. A One-Step Cone-Beam CT-Enabled Planning-to-Treatment Model for Palliative Radiotherapy – From Development to Implementation. Int J Radiat Oncol Biol Phys. 2012. 10.1016/j.ijrobp.2012.01.025. 10.1016/j.ijrobp.2012.01.025 22592043

[14] Siow T, Kim S. Pre-planning: A new approach to virtual simulation. J Radiother Pract. 2018. 10.1017/s1460396917000565.

[15] Le A, Stojadinovic S, Timmerman R (2018). Real-time whole-brain radiotherapy: A single-institution experience. Int J Radiat Oncol Biol Phys.

[16] MacPherson M, Montgomery L, Fox G, et al. On-line rapid palliation using helical tomotherapy: A prospective feasibility study. Radiother Oncol. 2008. 10.1016/j.radonc.2008.01.017. 10.1016/j.radonc.2008.01.017 18329119

[17] Dyer B, Nair C, Deardorff C, et al. Linear Accelerator-Based Radiotherapy Simulation Using On-Board Kilovoltage Cone-Mean Computed Tomography for 3-Dimensional Volumetric Planning and Rapid Treatment in the Palliative Setting. Technol Cancer Res Treat. 2019. 10.1177/1533033819865623. 10.1177/1533033819865623 PMC6676252 31370760

[18] Létourneau D, Wong R, Moseley D, et al. Online planning and delivery technique for radiotherapy of spinal metastases using cone-beam CT: image quality and system performance. Int J Radiat Oncol Biol Phys. 2007. 10.1016/j.ijrobp.2006.09.058. 10.1016/j.ijrobp.2006.09.058 17336223

[19] Wilson D, Sheng K, Yang W (2012). Natanasabapthi G. STAT RAD: a potential real-time radiation therapy workflow. Modern Practices in Radiation Therapy.

[20] Schuler T, Back M, Hruby G, et al. Introducing Computed Tomography Simulation-Free and Electronic Patient-Reported Outcomes-Monitored Palliative Radiation Therapy into Routine Clinical Care: Clinical Outcomes and Implementation Experience. Adv Radiat Oncol. 2021. 10.1016/j.adro.2020.100632. 10.1016/j.adro.2020.100632 PMC8039552 33851063

[21] Rich S, Chow R, Rama S (2018). Update of the systematic review of palliative radiation therapy fractionation for bone metastases. Radiother Oncol.

[22] Sekhon M, Cartwright M, Francis J. Acceptability of healthcare interventions: an overview of reviews and development of a theoretical framework. BMC Health Serv Res. 2017. 10.1186/s12913-017-2031-8. 10.1186/s12913-017-2031-8 PMC5267473 28126032

[23] Milat A, Lee K, Grunseit A, et al. Intervention Scalability Assessment Tool. Prepared by The Australian Prevention Partnership Center and NSW Ministry of Health. https://preventioncentre.org.au/wp-content/uploads/2019/11/The-ISAT-Oct-2019_FINAL.pdf

