# Supplementary material for: DART: diagnostic-CT-enabled planning: a randomized trial in palliative radiation therapy (study protocol)
Source: BMC Palliat Care. 2022 Dec 9;21:220. doi: 10.1186/s12904-022-01115-y (PMC9733349; doi:10.1186/s12904-022-01115-y)
Supplement: Supplementary file 1 — Additional file 1: Appendix A. Questionnaire Items Based on the Theoretical Framework of Acceptability. [file 12904_2022_1115_MOESM1_ESM.zip › Table from Appendix A - Questionnaire Items Based on TFAR3.docx]

| **TFA construct** | **Theoretical definition** | **Items (6) measuring patient stakeholders’ perceptions of acceptability** | **Items (9) measuring clinician stakeholders’ perceptions of acceptability** |
| --- | --- | --- | --- |
| **Affective attitude** | How an individual feels about the intervention | Overall, I feel that the steps needed to receive treatment today were:  1 – Unacceptable  2 – Slightly unacceptable  3 – Neither acceptable or unacceptable (neutral)  4 – Slightly acceptable  5 - Acceptable | I feel the dCT-enabled planning workflow, as an alternative to conventional CT simulation and planning, was:  1 – Unacceptable  2 – Slightly unacceptable  3 – Neither acceptable or unacceptable (neutral)  4 – Slightly acceptable  5 - Acceptable |
| **Burden** | The perceived amount of effort that is required to participate in the intervention | The amount of **effort** needed from me to receive my treatment today was:  1 – Unacceptable  2 – Slightly unacceptable  3 – Neither acceptable or unacceptable (neutral)  4 – Slightly acceptable  5 - Acceptable  The amount of **time** I needed to spend at the cancer centre today to receive my treatment was:  1 – Unacceptable  2 – Slightly unacceptable  3 – Neither acceptable or unacceptable (neutral)  4 – Slightly acceptable  5 - Acceptable | The amount of **effort** required of me to perform *my clinical duties* in the dCT-enabled planning workflow, compared to conventional CT simulation and planning, was:  1 – Much more effort  2 – Slightly more effort  3 – The same amount of effort  4 – Slightly less effort  5 – Much less effort  The amount of **time** required of me to perform *my clinical duties* in the dCT-enabled planning workflow, compared to conventional CT simulation and planning, was:  1 – Much more time  2 – Slightly more time  3 – The same amount of time  4 – Slightly less time  5 – Much less time |
| **Ethicality** | The extent to which the intervention has good fit with an individual’s value | n/a | n/a |
| **Intervention coherence** | The extent to which the participant understands the intervention, how it addresses their condition and how it works | I understand why I needed (Arm 1)/did not need (Arm 2) a CT scan appointment before my treatment today:  1 – Strongly disagree  2 – Disagree  3 – Neither agree or disagree (neutral)  4 – Agree  5 – Strongly agree | I understand the **rationale** of the dCT-enabled planning workflow:  1 – Strongly disagree  2 – Disagree  3 – Neither agree or disagree (neutral)  4 – Agree  5 – Strongly agree |
| **Opportunity costs** | The extent to which benefits, profits or values that must be given up to engage in the intervention | n/a | n/a |
| **Perceived effectiveness** | The extent to which the intervention is perceived as likely to achieve its purpose | n/a | The dCT-enabled planning pathway is a viable alternative to conventional CT simulation and planning for simple palliative radiation treatment:  1 – Strongly disagree  2 – Disagree  3 – Neither agree or disagree (neutral)  4 – Agree  5 – Strongly agree  It would be worthwhile to explore scaling up the dCT-enabled planning pathway to expand patient eligibility:  1 – Strongly disagree  2 – Disagree  3 – Neither agree or disagree (neutral)  4 – Agree  5 – Strongly agree  In my opinion, the **quality of** **care** offered to patients on the dCT-enabled planning pathway, compared to conventional CT simulation and planning, is:  1 – Much worse  2 – Worse  3 – Equivalent  4 – Better  5 – Much better  In my opinion, the **quality of** **radiation treatment** offered to patients on the dCT-enabled planning pathway, compared to conventional CT simulation and planning, is:  1 – Much worse  2 – Worse  3 – Equivalent  4 – Better  5 – Much better |
| **Self-efficacy** | The participant’s confidence that they can perform the behaviour(s) required to participate in the intervention | I knew and understood what was needed from me to receive treatment today:  1 – Strongly disagree  2 – Disagree  3 – Neither agree or disagree (neutral)  4 – Agree  5 – Strongly agree  I felt like I was able to do the things that my healthcare team asked me to do in order to get my treatment:  1 – Strongly disagree  2 – Disagree  3 – Neither agree or disagree (neutral)  4 – Agree  5 – Strongly agree | I am confident in my ability to complete the duties required of me *in my clinical role* in the dCT-enabled planning pathway:  1 – Strongly disagree  2 – Disagree  3 – Neither agree or disagree (neutral)  4 – Agree  5 – Strongly agree |
