# Supplementary figures and images for: DART: diagnostic-CT-enabled planning: a randomized trial in palliative radiation therapy (study protocol)
Source: BMC Palliat Care. 2022 Dec 9;21:220. doi: 10.1186/s12904-022-01115-y (PMC9733349; doi:10.1186/s12904-022-01115-y)

Please see attached file


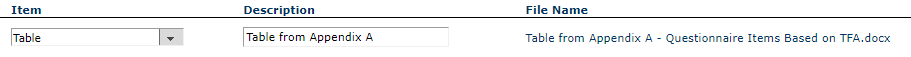

Supplement: Supplementary file 1 — Additional file 1: Appendix A. Questionnaire Items Based on the Theoretical Framework of Acceptability. [file 12904_2022_1115_MOESM1_ESM.zip › BMC DART_21Nov2022_APPENDIX AR3.docx]
